# Luteinising hormone-releasing hormone analogue reverses the cell adhesion profile of EGFR overexpressing DU-145 human prostate carcinoma subline

**DOI:** 10.1038/sj.bjc.6602350

**Published:** 2005-01-18

**Authors:** C Yates, A Wells, T Turner

**Affiliations:** 1 Department of Pathology, University of Pittsburgh, and Pittsburgh VAMC, Pittsburgh, PA 15261, USA; 2 Department of Biology and Center for Cancer Research, Carver Research Foundation, Tuskegee University, Tuskegee, AL 36088, USA

**Keywords:** tumour progression, EGF receptor, LHRH receptor, prostate cancer, cell adhesion

## Abstract

Cetrorelix, a luteinising hormone-releasing hormone (LHRH) analogue, has been shown to limit growth of the human androgen-independent prostate cell line DU-145, although other inhibitory actions may also be affected. Both growth and invasion of DU-145 cells are linked to autocrine epidermal growth factor receptor (EGFR) signalling. Invasiveness requires not only cells to migrate to conduits, but also reduced adhesiveness between tumour cells to enable separation from the tumour mass. Thus, we investigated whether Cetrorelix alters the DU-145 cell–cell adhesion and if this occurs via altered EGFR signalling. Pharmacologic levels of Cetrorelix limited the invasiveness of a highly invasive DU-145 subline overexpressing full-length EGFR (DU-145 WT). Extended exposure of the cells to Cetrorelix resulted in increased levels of the cell–cell adhesion complex molecules E-cadherin, *α*- and *β*-catenin, and p120. Puromycin blocked the increases in E-cadherin and *β*-catenin levels, suggesting that *de novo* protein synthesis is required. The Cetrorelix effect appears to occur via transmodulation of EGFR by a protein kinase C (PKC)-dependent mechanism, as there were no changes in DU-145 cells expressing EGFR engineered to negate the PKC transattenuation site (DU-145 A654); downregulation of EGFR signalling produced a similar upregulation in adhesion complex proteins, further suggesting a role for autocrine signalling. Cetrorelix increased the cell–cell adhesiveness of DU-145 WT cells to an extent similar to that seen when autocrine EGFR signalling is blocked; as expected, DU-145 A654 cell–cell adhesion also was unaffected by Cetrorelix. The increased adhesiveness is expected as the adhesion complex molecules moved to the cells' periphery. These data offer direct insight into the possible crosstalk pathways between the LHRH and EGFR receptor signalling. The ability of Cetrorelix to downregulate EGFR signalling and subsequently reverse the antiadhesiveness found in metastatic prostate cancer highlights a novel potential target for therapeutic strategies.

Adhesion between normal epithelial cells is usually strong and stable, limiting cell movement. In carcinomas, these tight cell associations must first be disrupted or prevented from forming before tumour cells are able to disseminate and metastasise. Cell–cell association is often disorganised in tumours, and has been linked to tumour invasiveness and metastasis (Pignatelli and Vessey, 1994; Shino *et al*, 1995; Richmond *et al*, 1997). Acquisition of invasive potential by malignant cancer cells results from an accumulation of characteristics, including increased cell motility, secretion of proteolytic enzymes, and alterations of cell–substrate and cell–cell adhesion (Fidler, 2003; Grunert *et al*, 2003). The molecular mechanisms responsible for this latter process, altered cell–cell adhesion, in invasive cancer cells are poorly understood (Comoglio and Trusolino, 2002). However, the net result is a reduction in cadherin/catenin complexes at the cells' periphery (Morita *et al*, 1999; Davies *et al*, 2000). Thus, to better understand the mechanisms of tumour cell dissociation, the role of cadherins must be taken into account, as they are crucial in cell–cell adhesion (Takeichi, 1993; Kim *et al*, 1999; Suyama *et al*, 2002).

Cadherins comprise a family of transmembrane cell surface glycoproteins that mediate calcium (Ca^2+^)-dependent, homotypic cell–cell interactions through their extracellular domains, and regulate a variety of biological processes during development, morphogenesis, and tumour metastasis (Gumbiner, 1996; Yap *et al*, 1997; Conacci-Sorrell *et al*, 2002). Ca^2+^-dependent cell–cell adhesion usually consists of rapid localisation of surface E-cadherin molecules to the regions of contact, resulting in homotypic binding that fosters the maintenance of normal cellular structure. However, metastatic cancer cells are able to over-ride or avoid contact inhibition signals employed by normal epithelial cells to control proliferation and cell movement.

The linkage between E-cadherin and the cellular cytoskeleton is a complex interaction involving a number of structural and signalling cytoplasmic proteins such as *α*- and *β*-catenin and p120 (Van Aken *et al*, 2001; Mason *et al*, 2002). Early studies identified E-cadherin/catenin interactions as imperative for cell–cell adhesion (Chitaev and Troyanovsky, 1998). *β*-Catenin binds with high affinity to the carboxyl-terminal region of the cadherin cytoplasmic tail, while *α*-catenin serves as an anchor, by bridging to *α*-actinin, to link the complex to the actin cytoskeleton (Aberle *et al*, 1994; Hulsken *et al*, 1994; Funayama *et al*, 1995; Jou *et al*, 1995; Rimm *et al*, 1995). These molecules not only play structural roles but also alter cell responses and phenotypes. *β*-Catenin is also found to immunoprecipitate with the APC tumour suppressor protein (Su *et al*, 1993; Hulsken *et al*, 1994; Shibata *et al*, 1994), and has been recently identified as an oncogene (Kim *et al*, 2002; Minamoto *et al*, 2002; Kielhorn *et al*, 2003; Schneider *et al*, 2003). It is also central to cell signalling, as upon dissociation from E-cadherin, it transits to the nucleus to alter transcriptional profiles (Mason *et al*, 2002; van de Wetering *et al*, 2002). A reduction in *β*-catenin expression decreases the stability of the adhesion complex and likely results in impairment in E-cadherin function (Willert and Nusse, 1998; Lowy *et al*, 2002; Mason *et al*, 2002). Similarly, a reduction in E-cadherin often results in *β*-catenin degradation (Liu *et al*, 2002). Another protein associated with E-cadherin, p120 (Thoreson *et al*, 2000), is phosphorylated on both tyrosine and serine residues in response to a variety of growth factors such as epidermal growth factor (EGF), platelet-derived growth factor (PDGF), and colony stimulating factor (CSF)-1, suggesting involvement in active signalling (Downing and Reynolds, 1991; Shibamoto *et al*, 1995). Thus, cell–cell adhesion serves not only a structural role but dictates cellular behaviour.

As carcinomas progress to the invasive and metastatic stages, select adhesive epithelial cells usually undergo a mesenchymal-like transition that enables their movement from the primary tumour mass (Comoglio and Boccaccio, 2001; Conacci-Sorrell *et al*, 2002). During this process in breast, gastric, and pancreatic metastatic carcinomas, E-cadherin expression is frequently downregulated or even undetectable (Birchmeier and Behrens, 1994; Lowy *et al*, 2002). This pattern of E-cadherin expression also persists in disseminated prostate carcinomas when compared to nonmetastatic prostate cells (Umbas *et al*, 1992; Davies *et al*, 2000; Mason *et al*, 2002). In addition, the loss of E-cadherin expression has been shown as a consequence of autocrine activation of epidermal growth factor receptor (EGFR) signalling (Jawhari *et al*, 1999). This combination of autocrine EGFR signalling and loss of E-cadherin expression leads to cell proliferation, dedifferentiation, and induction of cell motility (Hazan and Norton, 1998). Such an association has been suggested in the progression of breast carcinoma cells to a more invasive phenotype, which correlates with downregulation of E-cadherin and overexpression of EGFR (Sorscher *et al*, 1995a, 1995b; Hazan and Norton, 1998). On a molecular level, EGFR signalling leads to tyrosine phosphorylation of the catenin complex with subsequent breakdown of cell adhesion (Shiozaki *et al*, 1995; Jawhari *et al*, 1999; Mariner *et al*, 2004).

In this study, we examined whether the beneficial anticancer effects of Cetrorelix include effects in addition to the established antiproliferative effects. Luteinising hormone-releasing hormone (LHRH) receptors have increased expression in many cancers compared to normal cells (Emons *et al*, 1998; Schally *et al*, 2001; Straub *et al*, 2001), with increased expression in benign prostate hyperplasia (BPH) as well (Straub *et al*, 2003). The presence of these receptors enables LHRH analogues to affect directly prostate tumour cells (Qayum *et al*, 1990; Halmos *et al*, 2000) in addition to the indirect central androgen suppression. In addition, it has been shown that LHRH agonists directly inhibit cell proliferation of DU-145 and LNCaP prostate cancer cell lines (Dondi *et al*, 1994, 1998; Limonta *et al*, 2001). In line with these observations, the LHRH analogue Cetrorelix has been shown to have direct antiproliferative actions on DU-145 cells (Jungwirth *et al*, 1997b). As a consequence of this exposure, LHRH analogues have caused decreased levels of EGFR expression (Moretti *et al*, 1996; El-Bahrawy and Pignatelli, 1998; Lamharzi *et al*, 1998).

Previously, we have shown that DU-145 WT, a subline of the human prostate carcinoma cell line DU-145, presents autocrine EGFR signalling that is critical to both cell proliferation and invasion (Xie *et al*, 1995; Turner *et al*, 1996). Recently, we demonstrated under both *in vivo* and *in vitro* conditions that a LHRH agonist inhibited enhanced invasiveness of EGFR-dependent proliferation in DU-145 WT through interference with EGFR signalling (Wells *et al*, 2002). Therefore, these data taken together lead us to hypothesise that the LHRH analogue Cetrorelix would abrogate EGFR signalling. This abrogation would in turn decrease phosphorylation of the associated catenins, thus leading to upregulation of the cell adhesion molecule E-cadherin, which may ultimately result in inhibition of prostatic tumour progression.

## MATERIALS AND METHODS

### Materials

The LHRH analogue Cetrorelix ((Ac-D-Nal (2)^1^, D-Phe (4Cl)^2^, D-Pal (3)^3^, D-Cit^6^, D-Ala^10^) LH-RH) was obtained from ASTA Medica (Frankfurt/Main, Germany) and dissolved in serum-free Dulbecco's modified Eagle's medium (DMEM). The primary antibodies used were mouse monoclonal antibodies to E-cadherin, *α*- and *β*-catenin, and p120 (Transduction Laboratories, California, USA), phosphorylated MARCKS (Cell Signaling, Massachusetts, USA), phosphorylated EGFR (Cell Signaling, Massachusetts, USA), and EGFR (Zymed Laboratories, California, USA). FITC-conjugated secondary antibodies were obtained from BD Biosciences (California, USA). Secondary antibodies for the immunofluorescence were obtained from Molecular Probes (Oregon, USA). Inhibitors included the EGFR-specific tyrosine kinase inhibitor PD153035 (CalBiochem, California, USA), monoclonal antibody (528) EGFR (Oncogene, Massachusetts, USA), EGFR siRNA (Upstate, Virginia, USA), and the transcriptional and translational inhibitor puromycin (Sigma, Missouri, USA). Other reagents were obtained from Sigma.

### DU-145 cell lines

The cell line DU-145 was originally derived from a brain metastasis of a human prostate adenocarcinoma (Stone *et al*, 1978); it retains the androgen independence of the original tumour and does not express a functional androgen receptor (Dondi *et al*, 1998). This cell line possesses both LHRH and EGF receptors and produces EGFR ligands TGF-*α* and EGF (Xie *et al*, 1995; Jungwirth *et al*, 1997a). We have expressed exogenously encoded EGFR in DU-145 cells (Xie *et al*, 1995). Utilising established protocols, DU-145 cells were transfected by retroviral-containing EGFR constructs (Wells *et al*, 1990). The wild-type (WT) EGFR construct is a full-length cDNA derived from a placental cDNA library. Cells expressing WT EGFR at levels that escape downregulation demonstrate enhanced invasiveness *in vitro* (Xie *et al*, 1995) and *in vivo* (Turner *et al*, 1996).

The DU-145 WT subline expresses EGFRs that are phosphorylated and negatively modulated by protein kinase C (PKC); thus, we have generated an additional DU-145 subline that is not negatively modulated by PKC (Wells *et al*, 2002). This subline is identical to DU-145 WT except that it contains a full-length EGFR in which the target site for PKC phosphorylation, amino-acid threonine 654 (T654), has been replaced with alanine (DU-145 A654) by site-directed mutagenesis; this construct is resistant to PKC phosphorylation and negative transmodulation (Welsh *et al*, 1991; Chen *et al*, 1996).

The DU-145 WT and A654 cells were maintained in DMEM (4.5 g ml^−1^ glucose) (Cellgro, Virginia, USA) containing 10% FBS and supplemented with L-glutamine (2 mM), penicillin/streptomycin (100 U ml^−1^), nonessential amino acids (0.1 mM), and sodium pyruvate (1 mM) (37°C, 90% humidity, 5% CO_2_ and 95% air). For stable selection of WT or A654 EGFR, cells were grown in G418 (1000 *μ*g ml^−1^) (Gibco, New York, USA), although all experiments were performed in the absence of G418.

### Invasion assay

Cell invasiveness *in vitro* was determined by the ability of cells to transmigrate a layer of extracellular matrix, Matrigel, in a Boyden Chamber assay. Matrigel invasion chamber plates were obtained from Becton Dickinson Labware (Bedford, Massachusetts, USA). A total of 20 000 cells were plated in the Matrigel-containing chamber in serum-free media containing 1% BSA for the first 24 h; this was then replaced with Cetrorelix serum-free media for the remaining 24 h. Enumeration of the cells that invaded through the matrix over a 48 h period was accomplished by visually counting cells on the bottom of the filter. All experiments were performed in triplicate chambers.

### Flow cytometry

Cells (3 × 10^5^) were grown for 2 days or to 80% confluency in 60 mm plates. The LHRH analogue Cetrorelix (10^−5^ M) was added for time intervals of 6, 12, and 24 h and compared to diluent alone. Samples were washed with PBS and fixed with paraformaldehyde, and permeabilised with 1% Triton X-100. Samples were blocked with 5% BSA and incubated with the appropriate FITC-conjugated primary antibody or primary antibody (anti-EGFR, anti-E-cadherin, anti-*α*-catenin, anti-*β*-catenin, and anti-p120) at 37°C for 1 h. For unconjugated samples, FITC-conjugated secondary antibody was added. Fluorescence was measured by a flow cytometer (Coulter, Florida, USA).

### Immunoblotting

Cells (3 × 10^5^) were grown for 2 days or to 80% confluency in six-well plates. The LHRH analogue Cetrorelix (10^−5^ M) was incubated for 6, 12, and 24 h time intervals and compared to diluent alone. Protein lysates were prepared from cultured cells in the following buffer: 50 mM Tris, pH 7.5, 120 mM NaCl, 0.5% Nonidet P-40, 40 *μ*M phenylmethylsulphonylfluoride (PMSF), 50 *μ*g ml^−1^ leupeptin, and 50 *μ*g ml^−1^ aprotinin (all from Sigma). Cells were allowed to lyse for 1 h on ice; the lysed cell solution was centrifuged and the resulting supernatants were extracted and quantitated using a Bradford assay. Protein lysates (30 *μ*g) were separated by 7.5% SDS–PAGE, immunoblotted, and analysed using chemiluminescence (Amersham Biosciences, New Jersey, USA). Primary antibodies used included anti-EGFR (Zymed Diagnostics, California, USA), anti-E-cadherin, anti-*β*-catenin, and anti-p120 (Transduction Laboratories, Kentucky, USA), and anti-*α*-catenin (Santa Cruz Biotechnology, California, USA). The staining was visualised by a secondary anti-mouse IgG or anti-rabbit antibody linked to horseradish peroxidase (Promega, Wisconsin, USA).

### siRNA for EGFR

A total of 2 × 10^5^ cells were plated in six-well plates equalling 60–70% confluency. The EGFR siRNA (160 pmol) was diluted in 200 *μ*l of Opti-MEM (Invitrogen, California, USA). A 4 *μ*l portion of Lipofectamine 2000 (Invitrogen, California, USA) was diluted in 200 *μ*l of Opti-MEM and incubated for 5 min at room temperature. The diluted siRNA and Lipofectamine 2000 were mixed and incubated for 20 min at room temperature. Complexes were added to each well and incubated for 24 h. Media were changed and incubated for an additional 24 h. Cells were lysed according to established protocols.

### Immunofluorescence microscopy

A total of 3 × 10^5^ cells were grown for 2 days or to 80% confluency on glass coverslips and then treated with or without Cetrorelix (10^−5^ M) and compared to diluent alone. Cells were then fixed in 4% paraformaldehyde, permeabilised with 100 mM Tris-HCl pH 7.4, 150 mM NaCl, 10 mM EGTA, 1% Triton X-100, 1 mM PMSF, and 50 *μ*g ml^−1^ aprotinin (all from Sigma), and subsequently blocked with 5% BSA for 1 h at room temperature. Samples were incubated with indicated primary antibodies diluted in blocking buffer at 4°C overnight. FITC-conjugated secondary antibody was then added (BD Biosciences, California, USA). Cells were then stained with propidium iodine for nuclear staining. Cells were analysed with laser confocal microscopy using a Leica TCSNT 3 laser 4 PMT system (Olympus, New York, USA).

### Cell aggregation assay

Calcium-dependent aggregation of the DU-145 sublines was measured as previously described by Takeichi (1995) with the following modifications. Cell monolayers grown to 80% confluence were incubated for 24 or 48 h in 10% FBS in DMEM with or without 10^−5^ M Cetrorelix. Cell monolayers were detached from the culture dishes by incubating in cell stripper (Cell Gro, Virginia, USA) for 5–10 min at 37°C. Any remaining cells were detached using a rubber policeman, washed once with PBS, and collected by centrifugation. Cells were resuspended in 10% FBS in DMEM and single-cell suspensions made by trituration with a Pasteur pipette. Cell number was determined in the Coulter Counter Z1 (Coulter, Florida, USA). Cells were plated in triplicate wells of a 24-well plate at 2 × 10^5^ cells well^−1^ in 10% FBS in DMEM with 1 mM CaCl_2_ and allowed to aggregate for 60 min on a gyratory shaker at 80 r.p.m. at 37°C. Assays were stopped at 0 and 60 min by fixing the cells in 0.5% paraformaldehyde. The extent of cell–cell binding was monitored by measuring the disappearance of single cells using the Coulter Counter Z1. The index of the degree of aggregation was measured utilising the formula 100 × (*N*_0_/*N*_60_), where *N*_0_ is the total cell number per well and *N*_60_ is the total number of particles after 60 min of incubation as determined by counting in a Coulter Counter Z1.

### Statistical analysis

Statistics for all experiments were performed using the Sigma Plot statistical program (Jandel Scientific, California, USA). Independent Student's *t*-test was utilised to determine a statistical difference between experimental and the controls for individual experiments.

## RESULTS

### The LHRH analogue Cetrorelix decreases invasion in DU-145 sublines

To confirm and extend the inhibitory effects of Cetrorelix on prostate carcinomas, we utilised a genetically engineered human androgen-independent prostate carcinoma cell line that overexpresses a full-length EGFR, DU-145 WT. This subline is highly invasive in response to upregulation of autocrine EGFR signalling (Xie *et al*, 1995; Turner *et al*, 1996) that exists in practically all prostate carcinomas (Kim *et al*, 1999). In determining the utilised dose of Cetrorelix, we selected the pharmacologic dose of 10^−5^ M based on literature reports for Cetrorelix (Tang *et al*, 2002) and a related LHRH analogue goserelin (Dondi *et al*, 1994; Jungwirth *et al*, 1997a, 1997b; Limonta *et al*, 1998; Wells *et al*, 2002). In addition, growth studies from our laboratory utilising Cetrorelix at 10^−5^ M inhibited DU-145 WT proliferation without causing cell death (data not shown).

To probe the extent of effectiveness of Cetrorelix against prostate cancer progression, we determined whether invasion was abrogated. Cetrorelix exposure reduced the invasiveness of the DU-145 WT sublines through a Matrigel barrier from 100% down to 23±14% (Figure 6; *n*=4, *P*<0.05). This level of inhibition is comparable to the decreases noted when either EGFR motility signalling via PLC*γ* or calpain signalling is abrogated (Xie *et al*, 1995; Turner *et al*, 1996; Kassis *et al*, 1999; Mamoune *et al*, 2003).

### Cetrorelix increases levels of cell adhesion molecules

**Figure 1:**
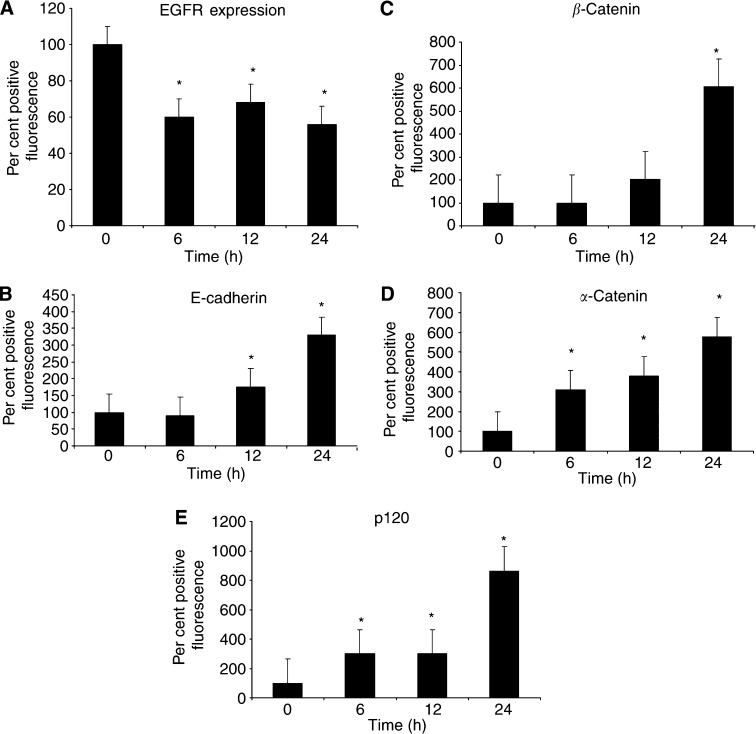
Expression levels were measured as the mean of per cent positive fluorescence at time zero±s.e.m. at various time intervals. (**A**) DU-145 WT cells labelled with FITC-conjugated anti-EGFR were analysed by flow cytometry. (**B**) DU-145 WT cells labelled with FITC-conjugated anti-E-cadherin were analysed by flow cytometry. (**C**) DU-145 WT cells labelled with FITC-conjugated anti-*β*-catenin were analysed by flow cytometry. (**D**) DU-145 WT cells labelled with FITC-conjugated anti-*α*-catenin were analysed by flow cytometry. (**E**) DU-145 WT cells labelled with FITC-conjugated anti-p120 were analysed by flow cytometry. Data are the mean±s.e.m. of three experiments each performed in triplicate. ^*^P<0.05 compared to untreated.

To determine the effectiveness of Cetrorelix treatment on altering protein expression levels, we measured EGFR, E-cadherin, and its associated adhesion molecules (*α*- and *β-*catenins, and p120) by flow cytometry. After 6 h of Cetrorelix exposure, EGFR levels were significantly reduced in DU-145 WT cells when compared to nontreated, control levels. This significant reduction in EGFR levels continued throughout the 24 h experimental time period (Figure 1A; *P*<0.05). While Cetrorelix decreased EGFR surface expression, it induced an increase in E-cadherin levels (Figure 1B). Likewise, the E-cadherin-associated molecules *α*-catenin, *β*-catenin, and p120 also demonstrated a continual increase in their expression, with all showing significant increases after 24 h of Cetrorelix exposure (Figure 1C–E; *P*<0.05%). To confirm results obtained from the flow cytometry experiments, we immunoblotted for whole-cell protein content of total EGFR and adhesion molecules E-cadherin and *β*-catenin. Again a similar pattern was seen, with a reduction in EGFR levels and an increase in E-cadherin and *β*-catenin levels (data not shown).

**Figure 2:**
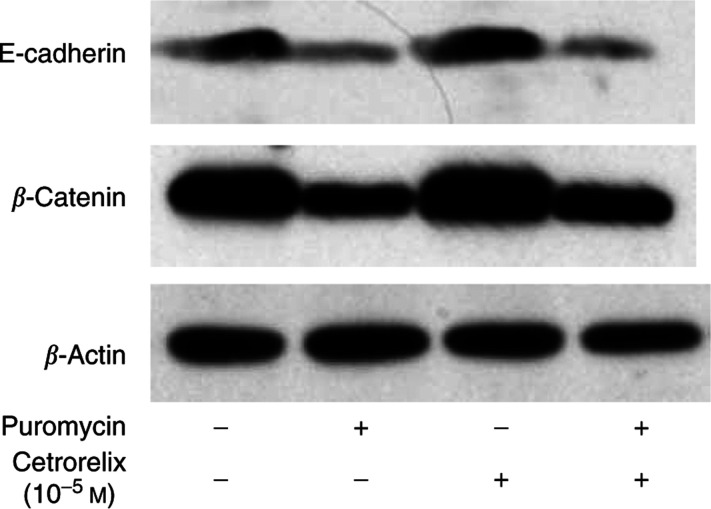
DU-145 WT cells were challenged with ±puromycin (40 *μ*M) in the presence of Cetrorelix (10^−5^ M) for 24 h. Lysates were collected and separated by SDS–PAGE, transferred, and immunoblotted with antibody to E-cadherin, antibody to *β*-catenin, or antibody to the loading control *β*-actin. Shown are representative blots of two experiments.

To thoroughly examine if the increases in protein and expression levels of E-cadherin and *β*-catenin were associated with upregulation in transcription, we used the protein synthesis inhibitor puromycin. Puromycin exposure was able to block completely the enhanced ability of Cetrorelix to restore the E-cadherin and *β*-catenin expression levels (Figure 2).

### Reversal in adhesion molecule profile is related to EGFR signalling

**Figure 3:**
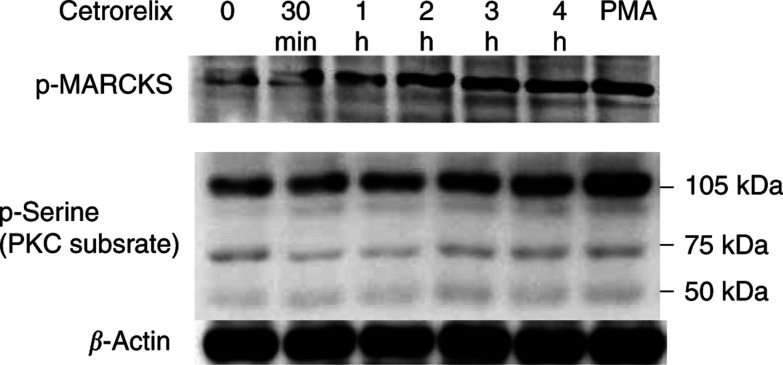
Top immunoblot: DU-145 WT cells were exposed to Cetrorelix (10^−5^ M) from 30 min to 4 h. Lysates were collected and separated by SDS–PAGE, transferred, and immunoblotted with antibody recognising phosphorylated MARCKS. Bottom immunoblot: DU-145 WT cells were challenged and immunoblotted with antibody recognising phosphorylated serine in the context of canonical PKC target sites. The bottom immunoblot demonstrates loading control of *β*-actin. Increases observed in the top two immunoblots are comparable to PMA-positive control. Shown are representative blots of three experiments.

A role for Cetrorelix in the stimulation of PKC activity was determined by phosphorylation of the MARCKS substrate for classical and novel PKC isoforms or by probing for generalised increased phosphorylation of canonical PKC target serines (Figure 3) (Fujise *et al*, 1994; Nishikawa *et al*, 1997). This was further confirmed through the use of chelerythrine, a pan-PKC inhibitor (Wells *et al*, 2002), to prevent such phosphorylation (data not shown).

**Figure 4:**
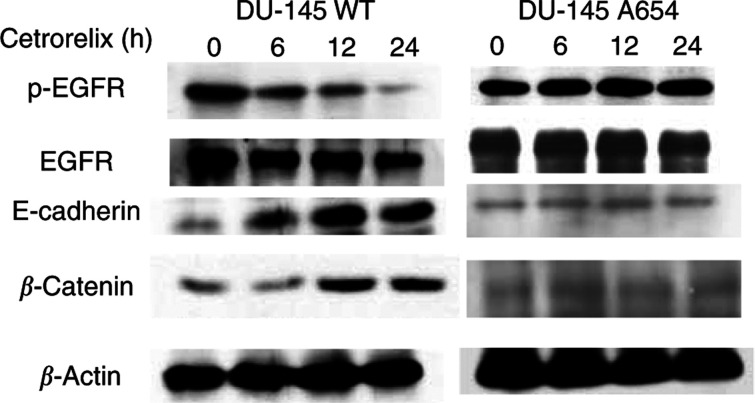
DU-145 WT (left immunoblots) and A654 (right immunoblots) cells were exposed to Cetrorelix (10^−5^ M) for up to 24 h. Lysates were collected and separated by SDS–PAGE, transferred, and immunoblotted with antibodies to p-EGFR, EGFR, E-cadherin, and *β*-catenin. Similar data were seen with *α*-catenin and p120 (data not shown). Shown are representative examples of three experiments.

**Figure 5:**
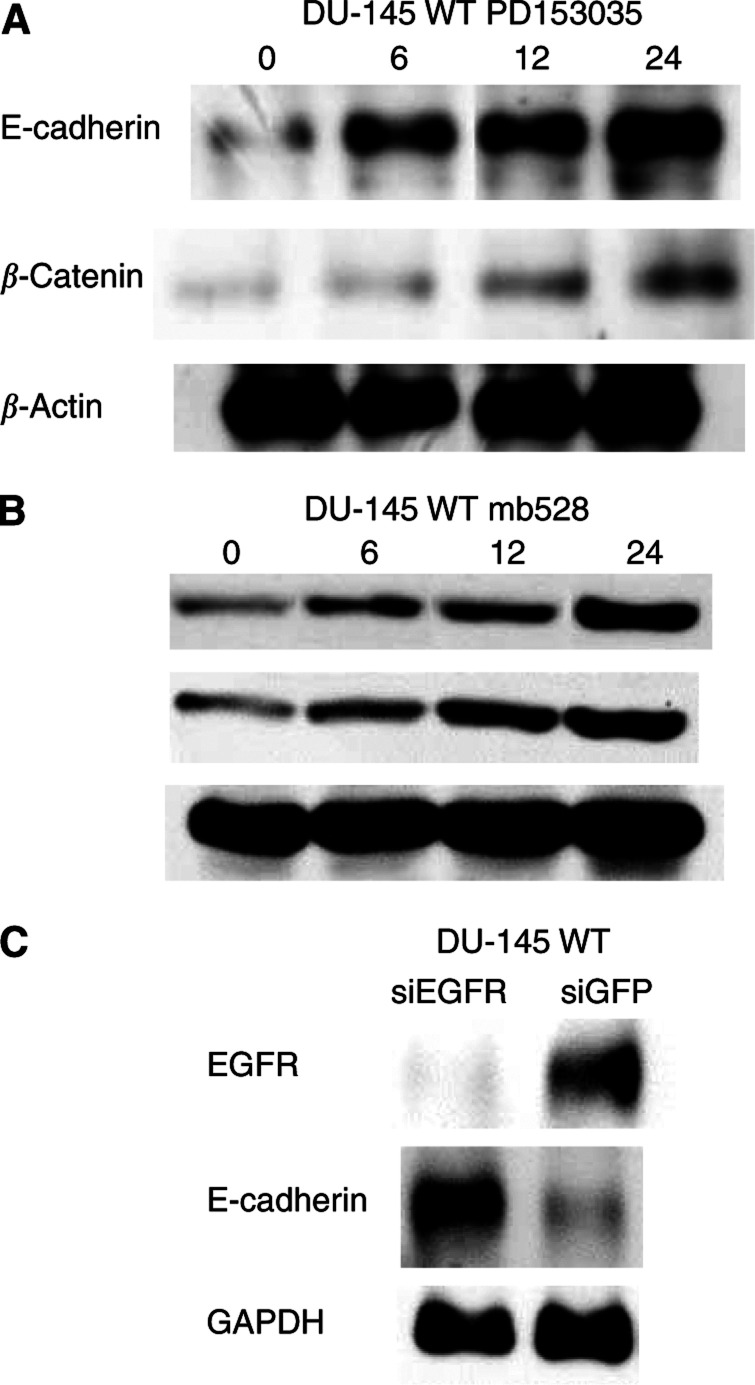
(**A**) DU-145 WT cells were exposed to PD153035 for 6, 12, and 24 h. (**B**) DU-145 WT cells were exposed to monoclonal antibody (528) against EGFR for 6, 12, and 24 h. (**C**) The EGFR siRNA was exposed to cells for 24 h and compared to the GFP siRNA. Lysates were collected and separated by SDS–PAGE, transferred, and immunoblotted with antibodies recognising EGFR, E-cadherin and *β*-catenin. Both *β*-actin and GAPDH were used as loading controls. One of two experiments for each point is shown.

If Cetrorelix acts via PKC-mediated attenuation of EGFR signalling, then an EGFR variant lacking the PKC target site should be resistant. We utilised a DU-145 subline expressing an EGFR construct in which the target PKC site, threonine 654, was replaced by an alanine (DU-145 A654). Since Cetrorelix decreased EGFR surface levels (Figure 1A) and increased surface levels and protein levels of cell adhesion molecules (Figures 1B–E and 2), cells expressing this EGFR A654 construct should be at least partly resistant to Cetrorelix. Through the use of immunoblotting techniques, we examined the protein levels of the cell adhesion molecules after 24 h of Cetrorelix exposure. Phosphorylated and total EGFR levels, as well as total E-cadherin and *β*-catenin levels were not extensively altered in the DU-145 A654 cells when compared to changes observed in DU-145 WT cells (Figure 4). These findings indicate that direct abrogation of EGFR signalling by various means should yield a similar increase in E-cadherin and *β*-catenin levels. Both the specific tyrosine kinase inhibitor PD153035 and the anti-EGFR antibody (mb528) increased E-cadherin and *β*-catenin levels similarly to those observed in DU-145 WT after Cetrorelix treatment (Figure 5A and B). Finally, exposure of DU-145 WT cells to EGFR siRNA resulted in the downregulation of EGFR levels and an increase in E-cadherin levels when compared to cells exposed to the nonrelevant siGFP (Figure 5C).

### Cetrorelix diminished prostate cancer cell invasiveness

**Figure 6:**
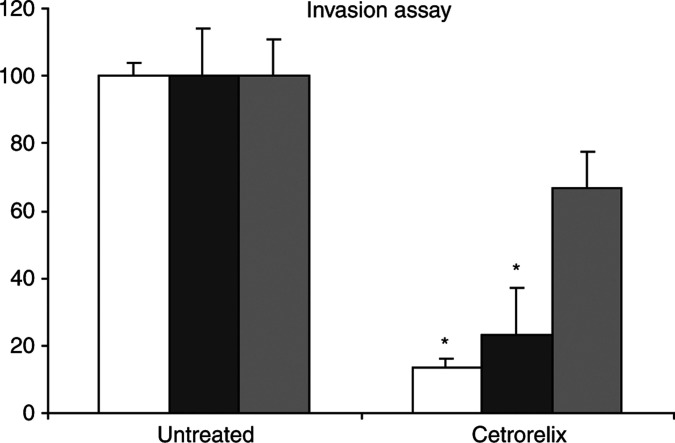
Cetrorelix reduced the invasiveness of the DU-145 parental (□) and DU-145 WT (▪) cells while only partly affecting that of DU-145 A654 (▒) cells. Invasiveness was measured by the cells' ability to transmigrate the extracellular matrix, Matrigel, in a Boyden Chamber assay. Data are the mean±s.e.m. (*n*=4). ^*^P<0.05, Cetrorelix-treated (48 h) groups *vs* controls (diluent only), without drug; also *P*<0.05 between the extent of decreased invasiveness of WT and A654 cells in the presence of Cetrorelix.

The functional consequences of EGFR signalling crossattenuation by Cetrorelix extend to the invasive potential of the prostate carcinoma cells. While Cetrorelix significantly reduced the invasiveness of the DU-145 parental and WT cells, the invasiveness of DU-145 A654 was limited to a lesser extent (Figure 6; *P*<0.05, comparing Matrigel invasion after Cetrorelix treatment of DU-145 A654 and WT cells). These findings suggest that the effects of Cetrorelix on both cell–cell adhesion molecules and cell invasiveness are mediated through its interference with the EGFR signalling cascade.

### Cetrorelix exposure increases cell–cell aggregation

**Figure 7:**
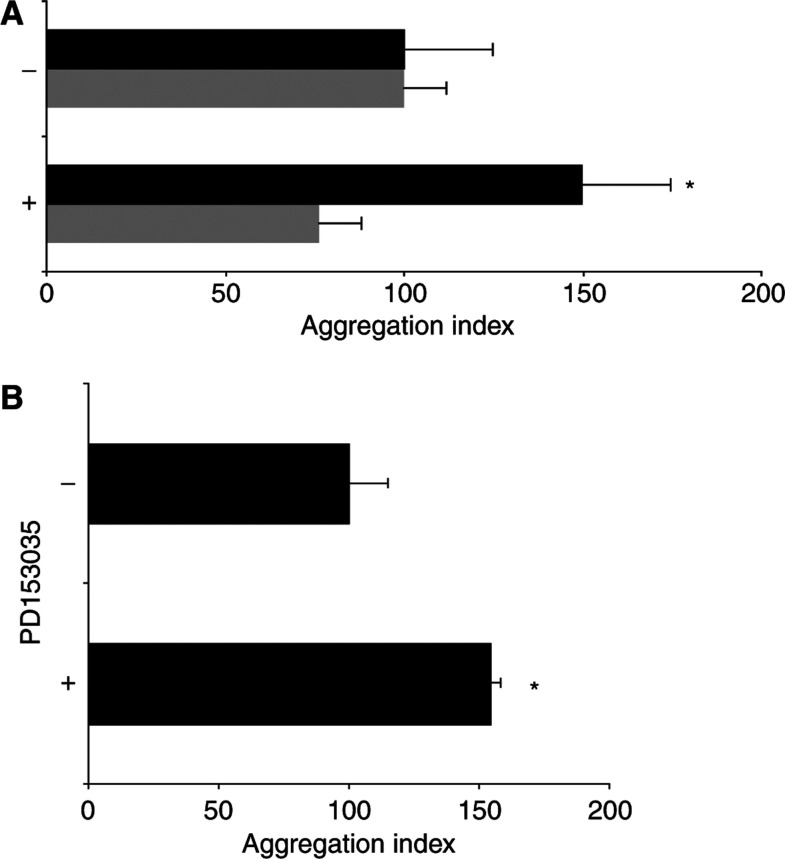
(**A**) Cetrorelix increased the cell–cell aggregation of the DU-145 WT (▪) cells after 48 h of exposure, while not affecting DU-145 A654 (▒) cells. (**B**) The EGFR inhibitor PD153035 increased the cell–cell aggregation of the DU-145 WT after 48 h of exposure. Results are expressed as the mean of the index of the degree of aggregation *vs* time zero±s.e.m. at 1 h (*n*=3, each in triplicate). ^*^P<0.05, Cetrorelix-treated (+) groups *vs* controls (−), without drug.

To further assess the functional consequences of the concurrent Cetrorelix-related decrease in EGFR levels and the increase in E-cadherin and its associated proteins observed in the DU-145 WT subline, a calcium-dependent aggregation assay was used after 48 h of Cetrorelix exposure (Figure 7). In these experiments, the aggregation index of DU-145 WT and A654 cells treated with Cetrorelix was compared to that of nontreated cells. We observed that DU-145 WT cells exposed to Cetrorelix formed significantly more cell–cell aggregates compared to either nontreated WT cells or treated and nontreated A654 cells, while Cetrorelix-induced DU-145 A654 aggregation was indistinguishable from nontreated cells (Figure 7; *P*<0.05). We were also able to see similar results when we exposed DU-145 WT cells to PD153035 to block EGFR signalling (Figure 7B; *P*<0.05%).

**Figure 8:**
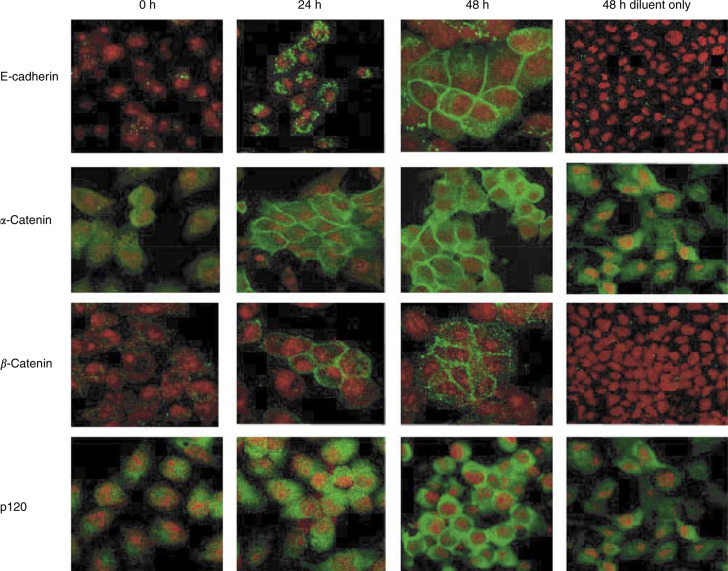
Cells were exposed to Cetrorelix for up to 48 h prior to immunofluorescent localisation of E-cadherin (top panels), *α*- and *β*-catenins (second and third panels, respectively), or p120 (bottom panels) and compared to 48 h diluent alone (right panel). Shown are representative photomicrographs of two independent experiments; the target molecules are green and nuclei are red.

Cell–cell aggregation requires E-cadherin to be present on the cell surface and its associate molecules at the inner face of the plasma membrane. In DU-145 WT cells, these adhesion complex molecules were distributed throughout the cytosol (Figure 8). Upon Cetrorelix treatment, not only did the levels increase, but also the molecules were redistributed to the cells' periphery; this was particularly evident at sites of cell–cell contacts, regardless of the degree of cell confluence. In aggregate, these data further confirmed with functional application that the increases observed in E-cadherin, *α*-and *β*-catenins, and p120 levels in Cetrorelix-exposed DU-145 WT cells are the results of a reversal of the cells invasive phenotype to one that resembles a more normal phenotype and that Cetrorelix exerts at least some of its effects via abrogation of autocrine EGFR cell signalling.

## DISCUSSION

The LHRH analogue Cetrorelix is undergoing evaluation for prostate cancer treatment. While initially considered for treatment due to its central androgen suppression mechanism, direct cancer cell efficacy has been shown. Cetrorelix has been demonstrated to limit proliferation of a variety of human cancer cell lines, including breast, ovarian, endometrial (Yap *et al*, 1997; Schally, 1999), and prostate cancer cell lines (Qayum *et al*, 1990; Halmos *et al*, 2000). Herein, we examined whether Cetrorelix altered an important phenotype of tumour cells, decreased cell–cell adhesion. We found that Cetrorelix exposure increased the levels of cell adhesion molecules and enhanced the resultant cell–cell adhesion. Furthermore, Cetrorelix appears to function, at least in part, by crossattenuation of signalling from the EGFR.

Several studies have long established that the loss of the homotypic E-cadherin binding machinery correlates with an invasive phenotype in prostate carcinomas (Behrens *et al*, 1989; Vleminckx *et al*, 1991; Bussemakers *et al*, 1992). Thus, it is logical that this cell–cell zipper would disappear concomitant with increased cellular invasion (Shibata *et al*, 1994). This disappearance of E-cadherin and/or any of the major adhesion components affiliated with it is noted in most advanced carcinoma cells (Takeichi, 1977; Hazan and Norton, 1998; Takeda *et al*, 1999). In fact, re-expression of E-cadherin has been shown to reduce the tumorigenicity of some carcinoma cell lines (Jawhari *et al*, 1999; Lowy *et al*, 2002). Interestingly, Cetrorelix exposure increases the levels of all of the major adhesion molecules probed; this could be secondary to either increased transcription or decreased degradation. This should subsequently lead to the reforming of the zipper. This was corroborated in our invasion (Figure 6) and aggregation studies (Figure 7A and B) where, after extended Cetrorelix exposure, the highly invasive WT cell line became less invasive and aggregated to a greater extent than nontreated cells.

The ability to exploit the findings that Cetrorelix treatment increases both cell–cell adhesion and the levels of the key molecules involved in the adhesion process is vastly improved by defining the underlying basis for this occurrence. Other LHRH analogues have been shown to limit prostate carcinoma cell growth secondary to downregulation of EGFR (Moretti *et al*, 1996; Jungwirth *et al*, 1997a, 1997b) or through interference with signalling pathways initiated by the EGFR (Wells *et al*, 2002). This occurred via PKC-mediated crossattenuation (Wells *et al*, 2002) secondary to phosphorylation on threonine 654 of EGFR (Lin *et al*, 1986; Welsh *et al*, 1991). In this study, we show direct activation of PKC substrate MARCKS by LHRH receptors in a time-dependent manner (Figure 3). These findings led us to believe that DU-145 cells engineered to express the PKC-resistant A654 EGFR should be impervious to Cetrorelix treatment. This was borne out by our findings that EGFR levels remained high and cell adhesion molecule levels low in these cells in the face of Cetrorelix exposure (Figure 4). The importance of EGFR signalling was further demonstrated in a time-dependent manner from the exposure of the DU-145 WT subline to an EGFR-specific tyrosine kinase inhibitor, PD153035, and a monoclonal antibody against EGFR (mb528) (Figure 5A and B). Cetrorelix and PD153035 both increased cell–cell adhesion in DU-145 WT, but had little effect on DU-A654 cells (Figure 7). The results of all of our findings taken together indicate that the ability of the LHRH analogue Cetrorelix to alter the adhesive profile of these cells is at least partly mediated through altered EGFR signalling.

That Cetrorelix restores cell–cell adhesion secondary to disrupting EGFR signalling would be supported if EGFR signalling could be shown to downregulate cell–cell adhesion. This was shown to occur at least in the DU-145 WT cells by their increased aggregation upon disruption of autocrine EGFR signalling (Figure 7). Epidermal growth factor receptor signalling, upregulated in an autocrine manner in prostate carcinomas (Kim *et al*, 1999), was shown to be responsible, at least in part, for the downregulation of cadherin-mediated adhesion and levels of molecules noted in these tumours as it is in many other carcinomas (Sorscher *et al*, 1995a, 1995b; Wilding *et al*, 1996; Jawhari *et al*, 1999; Andl *et al*, 2003). Another report indicated that EGFR downregulation resulted in decreasing E-cadherin and catenins in ovarian carcinoma cells (Alper *et al*, 2000). Although the reason for this opposite effect in these cells was not obvious, it may be related to the distinct nature of some ovarian cell types. Presumably, such a reduction in the levels of adhesion molecules plays a major role in prostate cancer progression (Wells, 2000). How EGFR signalling limits cadherin-mediated adhesions is still being deciphered (Ackland *et al*, 2003; Cozzolino *et al*, 2003). However, this appears to involve both acute phosphorylation of PKC and the dissociation and subsequent degradation of key adhesion components. Regardless of the actual mechanism, the end result is witnessed in the long-term downregulation of these molecules.

In summary, we found that Cetrorelix restored the adhesiveness of the human prostate carcinoma cells (and significantly inhibited cellular proliferation) at similarly high pharmacologic doses used by others (Jungwirth *et al*, 1997a; Tang *et al*, 2002). Additionally, the LHRH agonist Zoladex was shown to only inhibit *in vitro* cell proliferation of androgen-dependent (LNCaP) and androgen-independent (DU-145) cell lines at similarly high concentrations (Moretti *et al*, 1996; Wells *et al*, 2002). Thus it seems that higher concentrations of LHRH analogues are needed to accomplish direct cell growth inhibition than to achieve androgen suppression. There are obvious speculative reasons for this, but regardless of the mechanism, these studies serve as proofs of concepts that this signalling axis can be exploited to limit prostate tumour progression. It remains to be determined whether therapeutic interventions will exploit this using higher affinity analogues or indirect augmentation of the described pathway that crossattenuates the autocrine EGFR signalling pathway in tumour promotion.
